# High humidity reprograms the gut mycobiome to promote *Meyerozyma caribbica*–derived syringic acid and attenuate sepsis-induced acute kidney injury

**DOI:** 10.1128/msystems.01716-25

**Published:** 2026-06-15

**Authors:** Shumin Cai, Peiheng Guo, Hong Wang, Shanshan Fu, Kaixin Ding, Xiaoqi Liang, Ping Qin, Ze Wang, Yi He, Jie Li, Xiaoshan Zhao, Li Liu, Rong Wu

**Affiliations:** 1 Department of Health Management, Nanfang Hospital, Southern Medical University 70570, Guangzhou, China; 2 Department of Critical Care Medicine, Nanfang Hospital, Southern Medical University 70570, Guangzhou, China; 3 School of Traditional Chinese Medicine, Southern Medical University 70570, Guangzhou, China; 4 Medical Research Institute, Guangdong Provincial People's Hospital, Guangdong Academy of Medical Sciences, Southern Medical University 605403 , Guangzhou, China; 5 Department of Rheumatology and Immunology, The Third Affiliated Hospital of Southern Medical University 572489, Guangzhou, China; 6 Department of Neurology, The Third Affiliated Hospital of Southern Medical University 572489, Guangzhou, China; Chinese Academy of Sciences 85415 , Beijing, China

**Keywords:** sepsis-induced acute kidney injury, humidity, *Meyerozyma caribbica*, syringic acid

## Abstract

Sepsis-induced acute kidney injury (SAKI) remains a major contributor to mortality, yet the impact of environmental factors—particularly humidity—on disease progression is poorly understood. Here, we investigated how short-term high-humidity exposure shapes host susceptibility to SAKI and explored the underlying microbiota- and metabolite-mediated mechanisms. Mice pre-exposed to high humidity exhibited markedly attenuated renal injury and improved survival following cecal ligation and puncture (CLP). Notably, this protective effect persisted after bacterial depletion, but was abolished by amphotericin B treatment, indicating a fungus-dependent mechanism. Internal transcribed spacer sequencing and microbiota manipulation experiments identified *Meyerozyma caribbica* (*M. caribbica*) as a humidity-enriched commensal fungus essential for renal protection. Metabolomic profiling further revealed syringic acid (SA) as a key *M. caribbica*–derived metabolite responsible for the observed benefits. SA suppressed MAPK and NF-κB activation, reduced inflammatory cytokine release, and inhibited macrophage pyroptosis *in vitro*. Together, these findings demonstrate that high humidity confers protection against SAKI through an *M. caribbica*–SA axis that modulates macrophage inflammation and pyroptosis, highlighting a previously unrecognized environment–microbiota–host interaction in septic immunoregulation.

**IMPORTANCE:**

Sepsis outcomes are traditionally attributed to host immunity and microbial infection, whereas environmental influences remain largely overlooked. This study reveals that short-term environmental humidity profoundly shapes septic kidney injury through a commensal fungus–derived metabolite, establishing *M. caribbica* and its product syringic acid as key mediators of renoprotection. These findings challenge the conventional bacteria-centered view of sepsis–microbiota interactions and uncover humidity-driven mycobiota remodeling as a critical regulator of immune responses. By defining an environment–fungus–host axis that mitigates macrophage inflammation and pyroptosis, this work provides a conceptual framework for leveraging environmental modulation or fungal metabolites as novel therapeutic strategies for sepsis.

## INTRODUCTION

Sepsis is a severe clinical syndrome arising from a dysregulated host response to infection, resulting in systemic inflammation, immune dysfunction, and multi-organ failure (1). Globally, sepsis accounts for nearly 19.7% of all deaths, with sepsis-induced acute kidney injury (SAKI) affecting over 50% of acute kidney injury patients in intensive care units (ICUs), significantly increasing mortality rates (2, 3). SAKI is driven by microvascular collapse, metabolic disruption, and uncontrolled inflammation, yet interventions that effectively address these underlying mechanisms remain limited (3). Recent studies have begun to explore environmental factors, including ambient relative humidity, as potential modulators of infection risk and sepsis susceptibility (4–6). Low environmental humidity has been shown to increase influenza mortality by impairing mucociliary clearance and host barrier function and enhancing viral transmission (7, 8). However, whether humidity alters host susceptibility to bacterial sepsis or organ injury, particularly SAKI, has not been explored.

The human gut harbors a complex and dynamic microbiota that shapes host metabolism, immune homeostasis, and disease susceptibility (9). Although sepsis research has traditionally focused on bacterial communities (10), accumulating evidence indicates that fungal populations also serve as important immunomodulators (11). Gut mycobiome dysbiosis has been implicated in the progression of SAKI. For example, pathological expansion of fungi can promote systemic β-glucan translocation, which synergizes with bacterial endotoxins to exacerbate inflammatory injury (12). Clinical studies further reveal that septic and trauma patients hospitalized in the ICU for prolonged periods (2–3 weeks) exhibit marked mycobiome disruption, characterized by an overgrowth of opportunistic *Candida* species and a depletion of commensal genera such as *Penicillium* and *Saccharomyces* (13). Certain commensal species, such as gut-resident *Candida albicans*, produce metabolites such as phenylpyruvic acid that enhance macrophage bactericidal function and confer protection against sepsis (14). These findings suggest that fungal metabolites may represent previously underappreciated regulators of septic inflammation.

Among gut-resident fungi, *Meyerozyma caribbica* (*M. caribbica*) has recently gained attention due to its potential metabolic and probiotic functions (15). This fungus is generally regarded as non-pathogenic in healthy individuals and can be enriched under specific environmental conditions, yet its immunological relevance within the gut ecosystem remains largely unexplored. One phenolic metabolite associated with *M. caribbica* activity is syringic acid (SA), a hydroxybenzoic acid broadly distributed in medicinal plants (16) and produced by specific bacteria (17) as well as through fungal biotransformation (18). SA has been reported to exert cytoprotective and anti-inflammatory effects across diverse contexts (19–21), but whether SA participates in fungus–host interactions or contributes to immune regulation during sepsis has not been investigated.

In this study, we demonstrate that environmental humidity plays a critical role in modulating sepsis outcomes by reshaping the gut mycobiome and regulating host immune responses. Short-term exposure to high humidity enriches *M. caribbica*, a commensal yeast, which provides protection against SAKI. Metabolomic profiling reveals SA as a key fungal metabolite produced by *M. caribbica*. Mechanistically, SA inhibits MAPK, NF-κB, and NLRP3 inflammasome signaling in macrophages, suppressing inflammation and pyroptosis, and protecting against kidney injury. These findings uncover a humidity-driven fungal pathway that regulates immune responses, suggesting new therapeutic opportunities for sepsis through targeting the gut mycobiome.

## MATERIALS AND METHODS

### Mice

Eight- to ten-week-old specific pathogen-free male C57BL/6J mice were obtained from SiPeiFu Biotechnology Co., Ltd. (Beijing, China). All animals were housed under controlled environmental conditions (23 ± 2°C, 12-h light/dark cycle) with *ad libitum* access to food and water. Mice were acclimated for 1 week prior to experiments.

### Mouse model of cecal ligation and puncture (CLP)-induced sepsis

Mice were deeply anesthetized and placed supine on a sterile surgical table. Following disinfection and a 1–2 cm midline laparotomy, the cecum was carefully exteriorized and ligated 75% of its length distal to the ileocecal valve without disrupting blood flow. A through-and-through puncture was made at 1/3 of the ligated segment using an 18G needle, followed by gentle extrusion of a small amount of fecal content to ensure patency. The cecum was repositioned, and the peritoneum and skin were sutured. Mice received 1 mL of warm sterile saline subcutaneously for fluid resuscitation and were allowed to recover on a heating pad.

To investigate the effects of short-term high humidity exposure on sepsis outcomes, mice were pre-housed in artificial climate chambers (Chuanhong Experimental Instrument, Shanghai, China) at 23 ± 2°C under either normal humidity (NH, 55 ± 7%) or high humidity (HH, 90 ± 2%) for 7 days prior to CLP surgery. To evaluate the protective effect of *M. caribbica* (CICC 32557), mice were orally gavaged with *M. caribbica* suspension at a dose of 1 × 10^8^ CFU per mouse for 5 days before undergoing CLP surgery. To test the therapeutic role of SA (Macklin, Shanghai, China), mice were orally administered 100 mg/kg SA suspended in 0.5% CMC-Na for five consecutive days prior to CLP modeling. In all experiments, survival was monitored at 36 h post-CLP, and serum, kidney, and cecal tissues were collected 12 h post-CLP for downstream analyses.

### Cell culture and treatment

Bone marrow-derived macrophages (BMDMs) were isolated from the femurs and tibias of 8- to 12-week-old C57BL/6J mice and cultured at 37°C with 5% CO_2_ for 7 days in DMEM supplemented with 10% fetal bovine serum (FBS; Gibco, NY, USA), 1% penicillin–streptomycin (Gibco), and 20 ng/mL recombinant murine macrophage colony-stimulating factor (M-CSF; Miltenyi Biotec, Gladbach, Germany). Cells were seeded at a density of 5 × 10⁵ cells per mL for all experiments. For inflammatory cytokine analysis, BMDMs were pretreated with 10 μM SA (Macklin) for 2 h, followed by stimulation with 100 ng/mL LPS (Sigma, MI, USA) for 6 h. For immunoblotting assays, cells were pretreated with 10 μM SA for 30 min, then stimulated with 100 ng/mL LPS for 30 min. For pyroptosis induction, BMDMs were pretreated with 10 μM SA for 2 h, followed by LPS stimulation (500 ng/mL) for 4 h and subsequent ATP (5 mM; Sigma) treatment for 30 min.

### *M. caribbica* cultivation

*M. caribbica* was cultured in yeast malt (YM) medium containing yeast extract (Oxoid, UK), malt extract (Huankai Microbial, China), peptone, d-(+)-glucose, and agar (all from Sangon Biotech, China) at 28°C with shaking at 180 rpm for 2 days. Cells were harvested during logarithmic growth (OD_600_  = 0.8–1.2). Species identity was confirmed by sequencing and alignment using the NCBI BLASTN tool (https://blast.ncbi.nlm.nih.gov).

### Gut bacterial or fungal depletion and transplantation

To assess microbial contributions, mice were pretreated with a broad-spectrum antibiotic cocktail (ABX) consisting of metronidazole (100 mg/kg), neomycin sulfate (100 mg/kg), ampicillin (100 mg/kg), and vancomycin (50 mg/kg), administered by oral gavage for three consecutive days. This was followed by 7 days of humidity exposure, during which additional ABX doses were given on days 3 and 7. CLP surgery was performed on day 8. For fungal depletion, mice received 0.25 mg/mL amphotericin B (AMB) in drinking water throughout the 7-day humidity exposure prior to CLP. For fecal microbiota transplantation (FMT), recipient mice were pretreated with antibiotics for 3 days and subsequently gavaged with fecal suspensions (0.125 g/mL, 200 μL/day) derived from NH- or HH-exposed donors for five consecutive days. CLP was performed on day 6. All reagents were obtained from Macklin.

### Internal transcribed spacer (ITS) sequencing

Fecal fungal DNA was extracted using a Fungal DNA Mini Extraction Kit (Mabio, Guangzhou, China) according to the manufacturer’s instructions. DNA concentration and purity were assessed using an Epoch microplate reader (BioTek, Winooski, VT, USA). The ITS1/2 regions were amplified using barcode-tagged primers (ITS1F: 5′-CTTGGTCATTTAGAGGAAGTAA-3′; ITS1R: 5′-GCTGCGTTCTTCATCGATGC-3′; ITS2F: 5′-GCATCGATGAAGAACGCAGC-3′; and ITS2R: 5′-TCCTCCGCTTATTGATATGC-3′). Amplicons were pooled and sequenced on a PacBio platform. Bioinformatic analysis was performed to determine fungal diversity and relative abundance using high-performance computing platform.

### Metabolomic analysis

Non-targeted metabolomics was performed using a Vanquish UHPLC system coupled to an Orbitrap Q Exactive mass spectrometer (Thermo Scientific, USA). *M. caribbica* was cultured in YM medium at 28°C with shaking at 180 rpm for 48 h until OD_600_ ≈ 1. Culture supernatants and YM medium controls were collected by centrifugation at 8,000 rpm for 5 min at 4°C. An aliquot of each supernatant was mixed with an equal volume of methanol (vol/vol  =  1:1), vortexed, and centrifuged at 15,000 rpm for 15 min at 4°C. A 100 μL portion of the clarified supernatant was injected into a Hyperil Gold column (Thermo Scientific) for chromatographic separation. Elution was performed in positive ion mode using 0.1% formic acid (eluent A) and methanol (eluent B) with the following gradient: 0–1.5 min, 98% A; 1.5–3 min, 98–15% A; 3–10 min, 15–0% A; 10–10.1 min, 0–98% A; and 10.1–12 min, 98% A. The flow rate was set to 200 μL/min. Raw data were processed using Compound Discoverer 3.1 (Thermo Scientific).

### Liquid chromatography–tandem mass spectrometry (LC–MS/MS) analysis of SA

For *in vivo* colonization, mice were orally gavaged with *M. caribbica* (1 × 10^8^ CFU per mouse) or an equal volume of sterile PBS for five consecutive days. On day 5, fecal samples were collected. Cecal contents were weighed and extracted in methanol (1:9, wt/vol) by vortexing. For fungal metabolite preparation, culture supernatants were mixed with an equal volume of methanol (vol/vol = 1:1) and vortexed to precipitate proteins. All samples were centrifuged at 15,000 rpm for 15 min at 4°C and supernatants were vacuum-dried overnight. Dried samples were reconstituted in 200 μL methanol, sonicated on ice, and centrifuged again under the same conditions. For LC–MS/MS analysis, a 10 μL aliquot of the clarified supernatant was injected into a Shim-pack GIST C18-AQ HP column (Shimadzu, Tokyo, Japan). Chromatographic separation was performed on the Shimadzu LC system in positive-ion mode using 0.1% formic acid in water (mobile phase A) and methanol (mobile phase B). Samples were eluted with the following gradient: 10% B, 0–5 min; 10–100% B, 5–8 min; and 100% B, 8–10 min. The flow rate was maintained at 300 μL/min, and the total run time was 10 min. Data acquisition and processing were carried out using the Shimadzu software platform.

### Biochemistry and ELISA

Serum levels of creatinine (Cr) and blood urea nitrogen (BUN) were measured using commercial kits (Nanjing Jiancheng Bioengineering Institute, Nanjing, China). The cytokines IL-6, IL-1β, and TNF-α were quantified by ELISA (Neobioscience, Shenzhen, China) following the manufacturer’s instructions. Lactate dehydrogenase (LDH) released from BMDMs was quantified by using a CytoTox 96 Assay Kit (Promega, USA)

### Histopathology

Renal tissues were fixed in 4% paraformaldehyde (PFA) for 24 h, followed by dehydration, clearing, paraffin embedding, sectioning, and baking at 60°C for 1 h. Hematoxylin and eosin (H&E) staining was performed to assess histopathological alterations. Images were acquired under a Leica microscope. Renal injury was evaluated in a blinded manner using a semiquantitative scoring system based on five parameters: interstitial edema, epithelial alterations, tubular degeneration, capillary congestion, and leukocyte infiltration. Each parameter was graded on a scale of 0–4 according to injury severity. The score for each parameter was calculated as the mean value from multiple randomly selected fields per section, and the overall histopathological injury score was obtained by averaging the five parameter scores, as previously described (22).

### Quantitative reverse transcription PCR (qRT-PCR)

Total RNA from renal tissues or BMDMs was extracted using TRIzol reagent (Invitrogen, CA, USA). RNA quality was confirmed by OD_260/280_ ratio. Complementary DNA (cDNA) was synthesized using a reverse transcription kit (Toyobo, Osaka, Japan). qPCR was performed with SYBR Green Master Mix (Toyobo) on a Light Cycler 96 (Roche, Mannheim, Germany). Relative gene expression was normalized to *18s rRNA* and calculated using the 2^−ΔΔCt^ method.

The relative abundance of *M. caribbica* in fecal samples was calculated using the 2^−ΔCt^ method, where ΔCt = Ct_M_ − Ct_ITS_. The ITS1 region was used as an internal reference for normalization. Fold changes between groups were calculated as 2^−ΔΔCt^ = 2^− (ΔCt experimental − ΔCt control)^. Primers are listed in Table 1.

**TABLE 1:** Primers for qRT-PCR analysis

Primer	Forward (5′−3′)	Reverse (5′−3′)
*18S*	AGTCCCTGCCCTTTGTACACA	CGATCCGAGGGCCTCACTA
*Tnf-*α	CAGGCGGTGCCTATGTCTC	CGATCACCCCGAAGTTCAGTAG
*Il-6*	CTGCAAGAGACTTCCATCCAG	AGTGGTATAGACAGGTCTGTTGG
*Il-1*β	GAAATGCCACCTTTTGACAGTG	TGGATGCTCTCATCAGGACAG
*Il-18*	GACAGCCTGTGTTCGAGGATATG	TGTTCTTACAGGAGAGGGTAGAC
*Ccl2*	TAAAAACCTGGATCGGAACCAAA	GCATTAGCTTCAGATTTACGGGT
*Ccl3*	TGTACCATGACACTCTGCAAC	CAACGATGAATTGGCGTGGAA
*Cxcl2*	CCAACCACCAGGCTACAGG	GCGTCACACTCAAGCTCTG
*Nlrp3*	TCACAACTCGCCCAAGGAGGAA	AAGAGACCACGGCAGAAGCTAG
*ITS1*	CTTGGTCATTTAGAGGAAGTAA	GCTGCGTTCTTCATCGATGC
*M. caribbica*	AGATCAGACTCGATATTTTGTGAG	GTCTAGGCAGGCAGCATCAAC

### Western blot

BMDMs were lysed in RIPA buffer containing protease and phosphatase inhibitors (Beyotime, Shanghai, China), followed by heat denaturation in SDS loading buffer for 5 min. Equal amounts of protein were resolved by SDS–PAGE and transferred to PVDF membranes. After blocking with 5% BSA (Solarbio, Beijing, China), membranes were incubated overnight at 4°C with primary antibodies against caspase-1 (AdipoGen, USA), GSDMD (Abcam, USA), β-actin, p-JNK, JNK, p-ERK, ERK, p-p38, p38, p65, p-p65, and GAPDH (CST, USA). HRP-conjugated secondary antibodies (Huaan, Zhejiang, China) diluted 1:10,000 were used for detection, and signals were visualized using chemiluminescent substrate (Biosharp, Beijing, China). Band intensities were quantified using ImageJ.

### Fluorescence staining

Terminal deoxynucleotidyl transferase dUTP nick end labeling (TUNEL) staining assay was performed on paraffin-embedded tissue sections using a commercial kit (KeyGEN BioTECH, Nanjing, China) according to the manufacturer’s instructions. For visualization of apoptosis-associated speck-like protein containing a CARD (ASC) specks, BMDMs were fixed in 4% PFA for 15 min and permeabilized with 0.2% Triton X-100 for 10 min. After blocking nonspecific binding with 3% BSA for 30 min at room temperature, cells were incubated overnight at 4°C with an antibody against ASC (CST), followed by a 1-h incubation with Alexa Fluor 594-conjugated anti-rabbit IgG secondary antibody (CST). Nuclei were counterstained with Hoechst 33342 (ImmunoChemistry, MN, USA). For detection of cell death, BMDMs were stained with propidium iodide (PI) (ImmunoChemistry) for 10 min at 37°C.

### Statistical analysis

Data analysis was conducted using GraphPad Prism. For two-group comparisons, an unpaired Student’s *t*-test was employed. When more than two groups were compared, one- or two-way ANOVA followed by Sidak post hoc analysis was performed. Kaplan–Meier survival curves were analyzed using the log-rank test. Results are shown as mean ± SEM. Statistical significance was denoted as *P < 0.05, **P < 0.01, ***P < 0.001, and ****P < 0.0001.

## RESULTS

### Short-term high humidity exposure protects against sepsis-induced kidney injury

**Fig 1:**
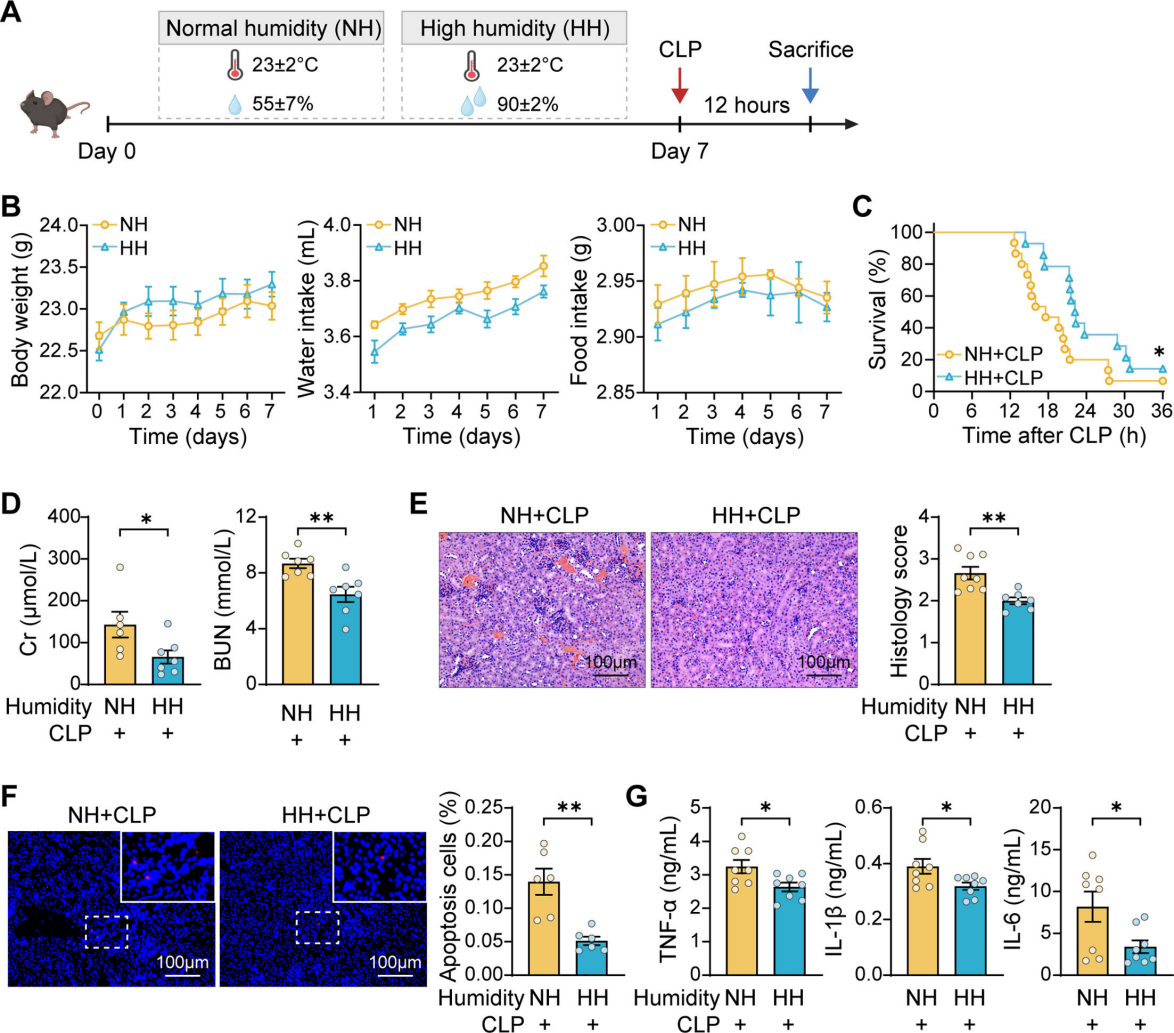
Short-term high-humidity exposure protects against sepsis-induced kidney injury. (**A**) Schematic diagram of the experimental design for high-humidity (HH) and normal-humidity (NH) exposure prior to CLP. (**B**) Body weight (*n* = 15), food intake (5 mice/cage, *n* = 3), and water consumption (5 mice/cage, *n* = 3) in mice exposed to NH or HH. (**C**) Survival curves of CLP-induced septic mice following short-term NH or HH exposure. *n* = 14 or 15. (**D**) Serum creatinine (Cr) and blood urea nitrogen (BUN) levels at 12 h post-CLP, *n* = 6 or 7. (**E**) Representative H&E staining and corresponding histopathological scores of kidney tissues. *n* = 7 or 8. (**F**) Representative TUNEL staining and quantification of apoptotic cells in kidney tissue. *n* = 6. (**G**) Serum TNF-α, IL-1β, and IL-6 concentrations determined by ELISA. *n* = 8. Data are presented as mean ± SEM. Statistical analyses were performed using two-tailed unpaired Student’s *t*-test (**D–G**) or two-way ANOVA with Sidak *post hoc* tests (**B**). Survival curves were analyzed using the Kaplan–Meier method with the log-rank test (**C**). *P < 0.05 and **P < 0.01. Scale bars, 100 μm.

To assess whether environmental humidity influences susceptibility to sepsis, mice were pre-exposed to high humidity (HH; 90% ± 2%) or normal humidity (NH; 55% ± 7%) at 23 ± 2°C for 7 days before undergoing CLP (Fig. 1A). There were no significant differences in body weight, water consumption, or food intake between groups prior to surgery (Fig. 1B). Following CLP, high humidity preconditioning significantly increased survival compared with NH-exposed mice (Fig. 1C). Biochemical analysis at 12 h post-CLP revealed that serum Cr and BUN levels were markedly lower in HH-exposed mice, indicating attenuated renal dysfunction (Fig. 1D). Histological assessment further showed that high humidity exposure alleviated tubular injury, reduced inflammatory infiltration, and preserved glomerular integrity (Fig. 1E). Consistently, TUNEL staining demonstrated fewer apoptotic cells in renal tissues of HH-exposed mice compared with NH controls (Fig. 1F). Moreover, ELISA results revealed significantly decreased levels of TNF-α, IL-1β, and IL-6 in HH-exposed mice (Fig. 1G). Collectively, these data indicate that short-term high humidity exposure alleviates SAKI and systemic inflammation, thereby improving survival outcomes.

### Intestinal fungi mediate the protective effects of high humidity in septic mice

**Fig 2:**
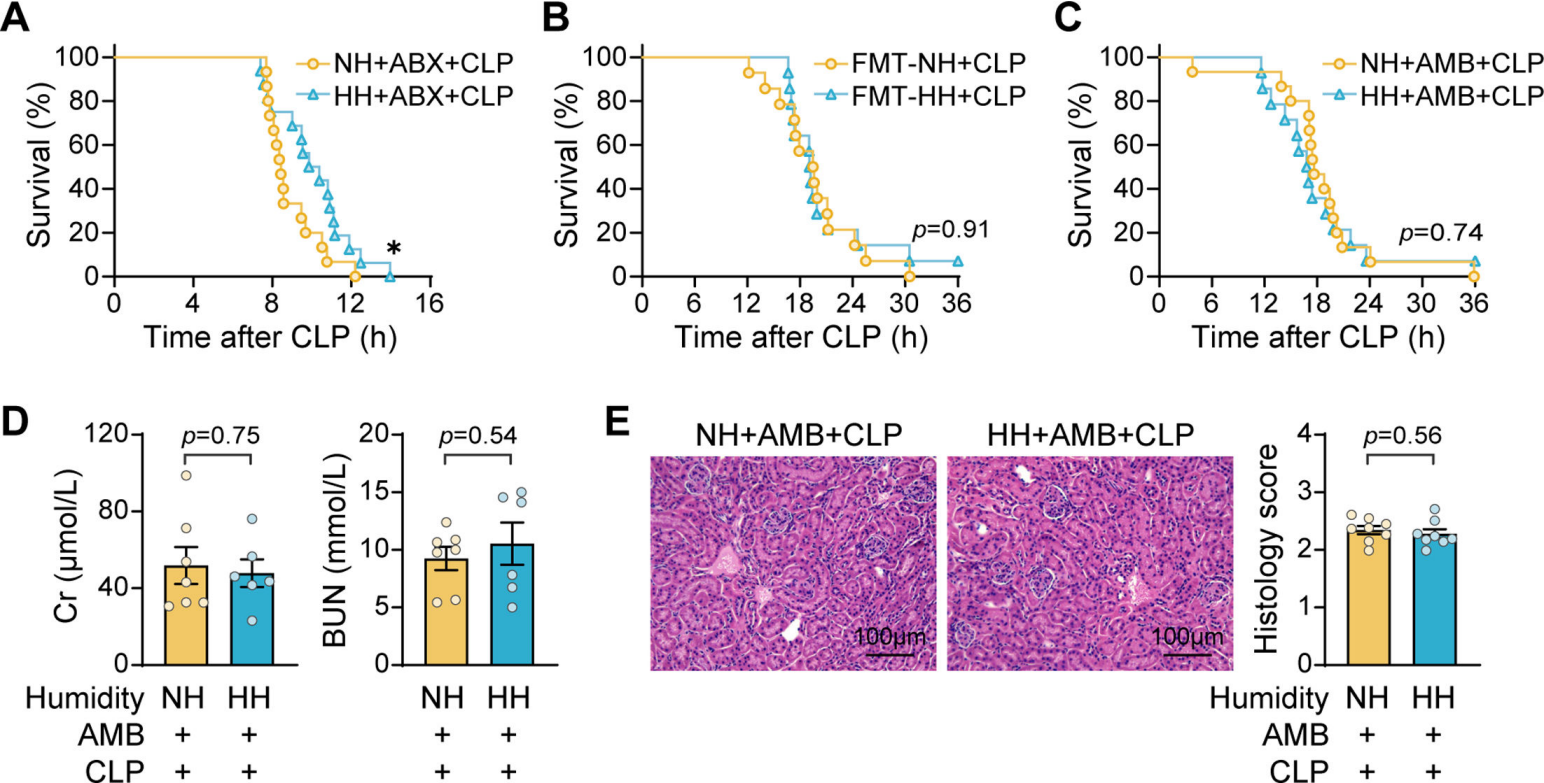
Gut fungi are required for the protective effects of high humidity exposure in sepsis. (**A**) The survival curve of CLP-induced septic mice co-treated with antibiotics (ABX) and short-term NH or HH exposure. *n* = 15 or 16. (**B**) The survival curve of ABX-treated mice receiving fecal microbiota transplantation (FMT) from NH- or HH-exposed donors followed by CLP. *n* = 14. (**C**) The survival curve of CLP-induced septic mice treated with amphotericin B (AMB) under NH or HH conditions. *n* = 14 or 15. (**D**) Serum Cr and BUN levels in septic mice treated with AMB under NH or HH exposure. *n* = 6 or 7. (**E**) Representative H&E staining and corresponding histopathological scores of kidney tissue from septic mice co-treated with AMB under NH or HH exposure. *n* = 8. Data were represented as mean ± SEM. The survival rates of septic mice were analyzed using the Kaplan–Meier method with the log-rank test (**A–C**). Statistical comparison was performed by two-tailed unpaired Student’s *t*-test (**D and E**). *P < 0.05. Scale bars: 100 μm.

Gut microbiota plays a critical role in modulating host responses during sepsis. To assess whether the protective effects of high humidity are dependent on gut bacteria, we generated pseudo-germ-free mice by administering ABX for 3 days. Remarkably, despite bacterial depletion, high humidity preconditioned mice still exhibited a significant survival advantage compared with NH controls after CLP (Fig. 2A), indicating that bacterial communities are not required for high humidity-mediated protection. Consistently, FMT from HH- or NH-exposed donors into antibiotic-treated recipients failed to reproduce the survival benefit, further supporting a non-bacterial mechanism (Fig. 2B).

We next investigated the potential involvement of intestinal fungi using the antifungal agent AMB during high humidity exposure. AMB administration completely abolished the survival advantage observed in HH-exposed septic mice (Fig. 2C). In parallel, serum Cr and BUN levels showed no significant differences between HH + AMB and NH + AMB mice (Fig. 2D), and histological analysis revealed persistent tubular damage and inflammatory infiltration in the kidneys of HH + AMB mice (Fig. 2E). Together, these findings—supported by ABX, FMT, and AMB experiments—demonstrate that intestinal fungi, rather than bacteria, are indispensable mediators of the protective effects of high humidity exposure in septic mice.

### High humidity promotes *M. caribbica*–derived SA production

**Fig 3:**
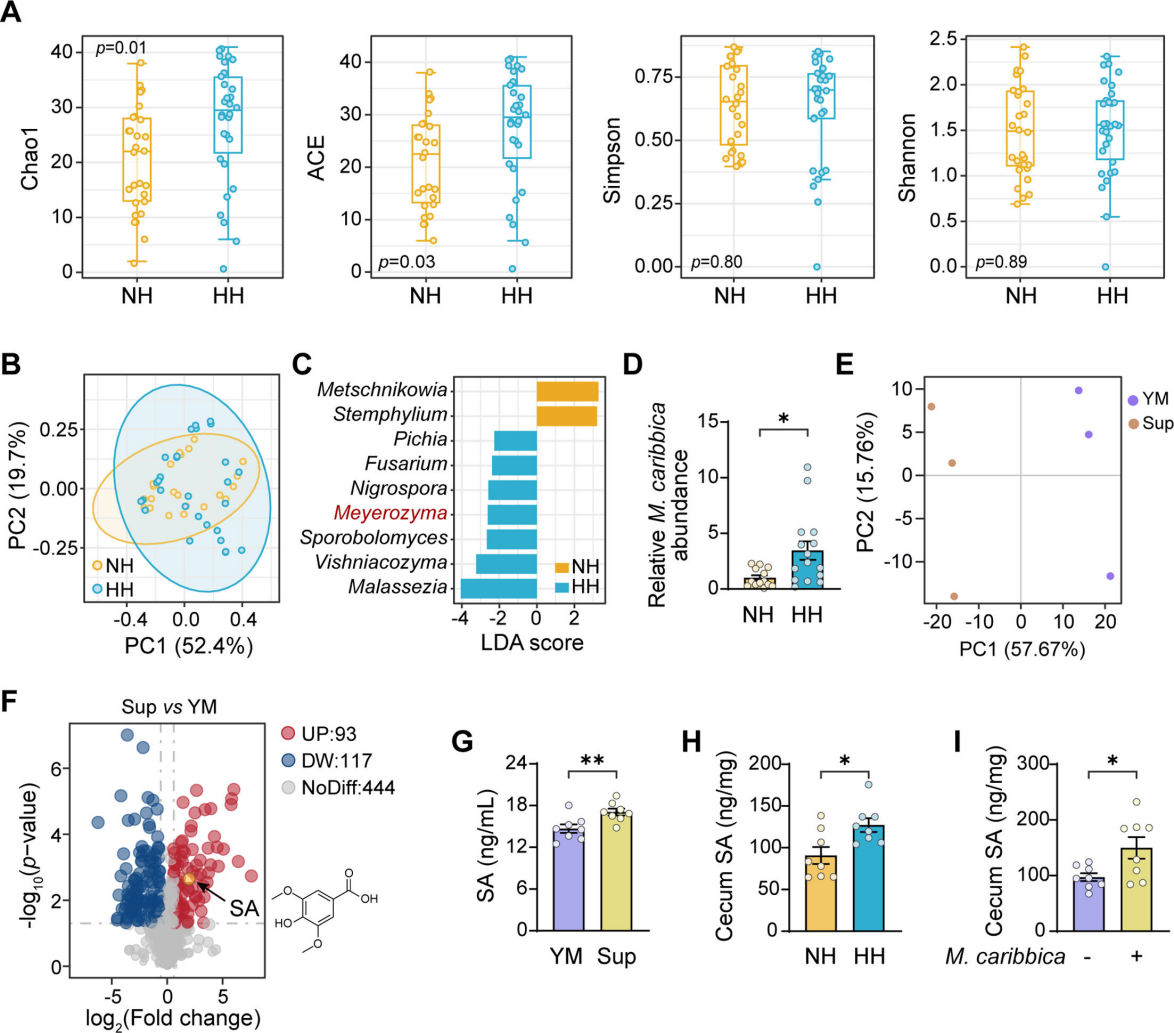
High humidity exposure enriches *M. caribbica* and promotes SA production. (**A**) Alpha diversity of gut fungal communities in mice subjected to short-term NH or HH exposure. Diversity indices include Shannon, Simpson, ACE, and Chao1. *n* = 23–25. (**B**) Principal coordinate analysis (PCoA) of gut fungal communities based on Bray–Curtis distance. *n* = 23–25. (**C**) Linear discriminant analysis effect size (LEfSe) analysis showing differentially enriched fungal genera between NH- and HH-exposed mice. *n* = 23–25. (**D**) Relative abundance of *M. caribbica* in fecal samples from NH- or HH-exposed mice. *n* = 13–15. (**E**) Principal component analysis (PCA) of metabolites from *M. caribbica* culture supernatants (Sup) versus yeast malt (YM) medium. *n* = 3. (**F**) Volcano plot of differentially abundant metabolites (VIP scores > 1, fold change >1.5 or < 0.667, *P* < 0.05) between *M. caribbica* and YM medium. *n* = 3. (**G**) Quantification of SA in *M. caribbica* culture supernatants and YM medium measured by LC–MS/MS. *n* = 8. (**H**) SA concentrations in cecal contents of NH- or HH-exposed mice measured by LC–MS/MS. *n* = 8. (**I**) SA concentrations in cecal contents of mice colonized with *M. caribbica* or PBS controls measured by LC–MS/MS. *n* = 8. Data were represented as mean ± SEM. Statistical comparison was performed by two-tailed unpaired Student’s *t*-test (**G–I**). *P < 0.05 and **P < 0.01.

To identify fungal taxa associated with the protective phenotype observed under high humidity, we performed ITS sequencing on fecal samples from HH- and NH-exposed mice. While Shannon and Simpson indices showed no difference, HH-exposed mice exhibited significantly higher fungal richness based on Chao1 and ACE metrics (Fig. 3A). Principal coordinate analysis (PCoA) based on Bray–Curtis distance revealed distinct clustering between HH and NH groups (Fig. 3B). Linear discriminant analysis Effect Size (LEfSe) identified *Malassezia*, *Vishniacozyma*, *Sporobolomyces*, and *Meyerozyma* as genera enriched under high-humidity conditions (Fig. 3C). Among these, *Meyerozyma*—a yeast genus with documented probiotic properties—is notable for its reduction in inflammatory conditions such as colitis (23). qRT-PCR confirmed a significant increase in *M. caribbica* abundance in the fecal microbiota of HH-treated mice (Fig. 3D).

To investigate the metabolic profile of *M. caribbica*, untargeted metabolomics was performed on the culture supernatants, with sterile YM medium serving as the blank control. Principal component analysis (PCA) revealed a pronounced divergence in global metabolite composition (Fig. 3E). We identified 210 differentially abundant metabolites, of which 93 were significantly enriched and 117 were depleted in *M. caribbica* (Fig. 3F and Table S1). To prioritize candidates for downstream validation, we considered statistical significance and effect size together with annotation confidence, biological relevance to host protection, and feasibility for targeted quantification using authentic standards. SA was significantly enriched in the untargeted data set and met these prioritization criteria, and was therefore selected for targeted verification. To determine whether *M. caribbica* directly produces SA, we analyzed fungal culture supernatants using LC–MS/MS. As expected, SA concentrations were markedly elevated in *M. caribbica* culture supernatants (Fig. 3G). Consistently, SA levels were significantly higher in the cecal contents of mice exposed to high humidity and in those colonized with *M. caribbica* (Fig. 3H and I). Together, these findings identify *M. caribbica* as a humidity-enriched commensal yeast capable of producing the bioactive metabolite SA.

### *M. caribbica* mitigates sepsis-induced kidney injury

**Fig 4:**
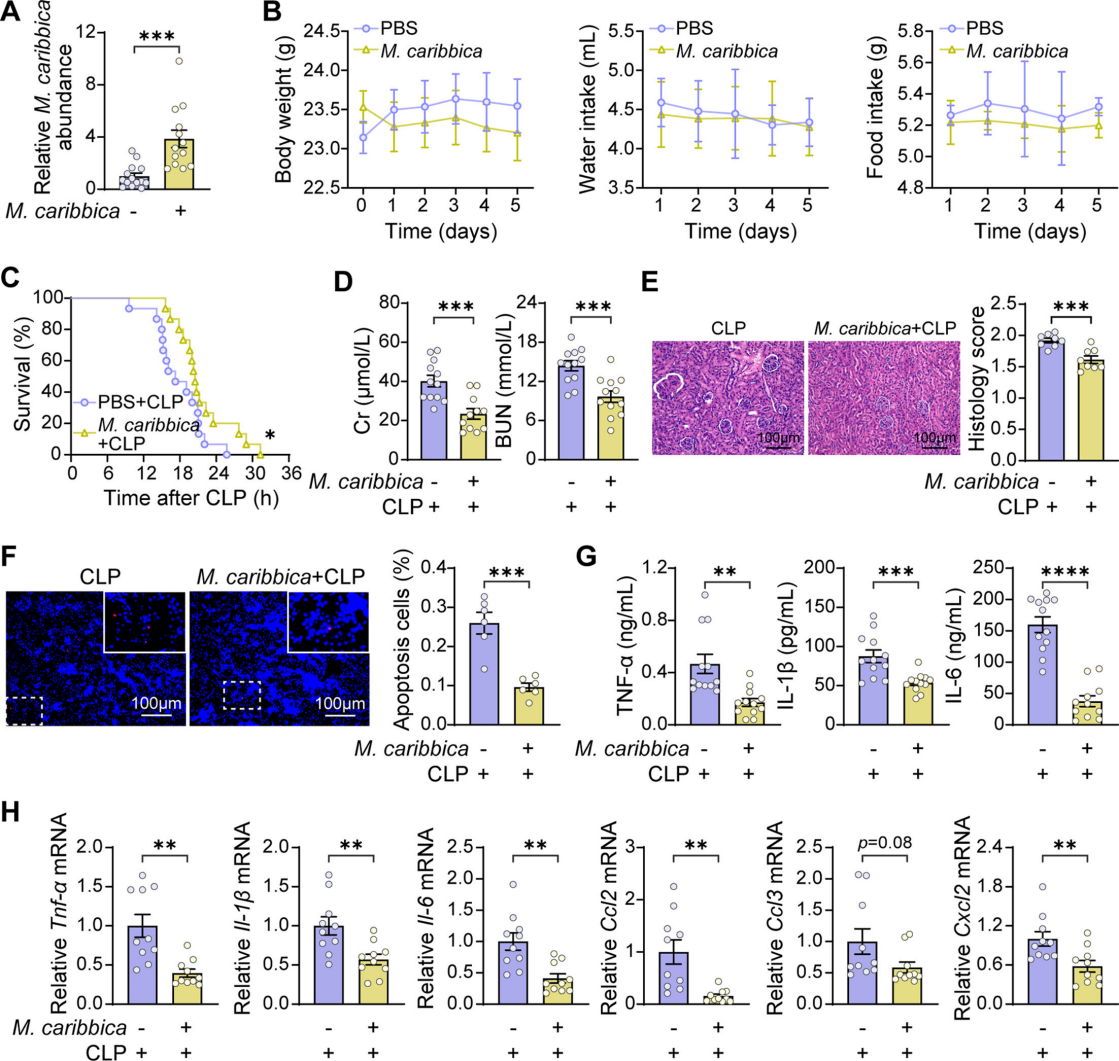
*M. caribbica* alleviates kidney injury and inflammation in septic mice. (**A**) Relative fecal abundance of *M. caribbica* after 5-day oral administration. *n* = 13 or 14. (**B**) Body weight (*n* = 15), food intake (5 mice/cage, *n* = 3), and water consumption (5 mice/cage, *n* = 3) in mice treated with or without *M. caribbica*. (**C**) Survival curves of CLP-induced septic mice pretreated with or without *M. caribbica*. *n* = 15. (**D**) Serum Cr and BUN levels in septic mice. *n* = 11 or 12. (**E**) Representative H&E staining and histopathological scoring of kidney tissues. *n* = 8. (**F**) TUNEL staining and quantification of apoptotic cells in kidney sections. *n* = 6. (**G**) Serum TNF-α, IL-1β, and IL-6 concentrations determined by ELISA. *n* = 11 or 12. (**H**) qRT-PCR analysis of *Tnf-*α, *Il-1*β, *Il-6*, *Ccl2*, *Ccl3*, and *Cxcl2* mRNA levels in kidney tissues. *n* = 10. Data were represented as mean ± SEM. Statistical analyses were performed using two-tailed unpaired Student’s *t*-test (**A, D–H**) or two-way ANOVA with Sidak *post hoc* tests (**B**). Survival curves were analyzed using the Kaplan–Meier method with the log-rank test (**C**). *P < 0.05, **P < 0.01, ***P < 0.001, and ****P < 0.0001. Scale bars: 100 μm.

To determine the functional contribution of *M. caribbica*, mice were orally administered 1 × 10^8^ CFU/mL of the yeast daily for five consecutive days. This regimen resulted in stable colonization, as evidenced by a significant elevation in fecal abundance compared with PBS-treated controls (Fig. 4A), without affecting body weight, water consumption, or food intake (Fig. 4B). Following CLP challenge, *M. caribbica*-treated mice exhibited prolonged survival (Fig. 4C), reduced serum Cr and BUN levels (Fig. 4D), and markedly attenuated kidney injury, characterized by preserved tubular architecture and reduced inflammatory cell infiltration (Fig. 4E). TUNEL assays further revealed a significant reduction in kidney apoptotic cell death (Fig. 4F). *M. caribbica* administration suppressed systemic levels of TNF-α, IL-1β, and IL-6 (Fig. 4G). Consistently, qRT-PCR analysis of kidney tissue revealed significant reductions in *Tnf-*α, *Il-1*β, *Il-6*, *Ccl2*, and *Cxcl2*, while *Ccl3* showed a non-significant downward trend (Fig. 4H). Collectively, these results identify *M. caribbica* as a key fungal species enriched by high humidity and demonstrate its capacity to confer protection against sepsis-induced kidney injury.

### SA mediates the renoprotective effects of *M. caribbica* in sepsis

**Fig 5:**
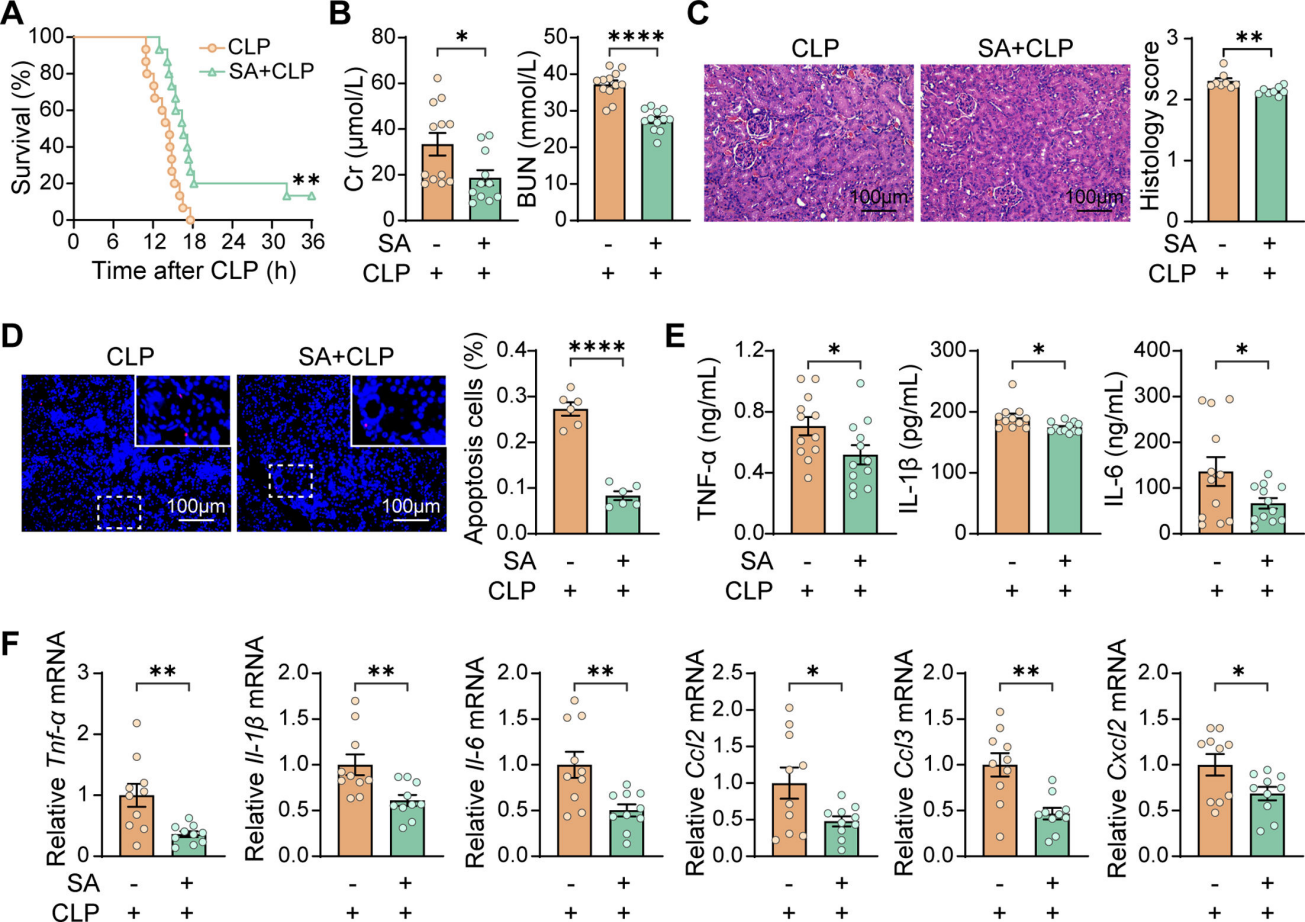
SA attenuates kidney injury and systemic inflammation in septic mice. (**A**) Survival curves of CLP-induced septic mice pretreated with or without SA. *n* = 15. (**B**) Serum Cr and BUN levels in septic mice pretreated with or without SA. *n* = 11 or 12. (**C**) Representative H&E staining and histopathological scoring of kidney tissues in septic mice pretreated with or without SA. *n* = 8. (**D**) TUNEL staining and quantification of apoptotic cells in kidney sections. *n* = 6. (**E**) ELISA quantification of serum TNF-α, IL-1β, and IL-6 levels in septic mice. *n* = 11 or 12. (**F**) qRT-PCR analysis of pro-inflammatory cytokine and chemokine mRNA levels in kidney tissues from septic mice. *n* = 10. Data were represented as mean ± SEM. Survival curves analyzed using the Kaplan–Meier method with the log-rank test (**A**). Statistical comparison was performed by two-tailed unpaired Student’s *t*-test (**B–F**). *P < 0.05, **P < 0.01, and ****P < 0.0001. Scale bars: 100 μm.

To evaluate whether SA accounts for the protective effects of *M. caribbica*, mice were pretreated with SA or vehicle control via oral gavage prior to CLP surgery. SA administration resulted in a significant extension of survival time compared to vehicle-treated controls (Fig. 5A), accompanied by markedly lower serum Cr and BUN levels (Fig. 5B). Histopathological examination revealed substantial attenuation of renal tubular damage and inflammatory cell infiltration in SA-treated mice relative to controls (Fig. 5C). Consistent with these findings, TUNEL staining demonstrated reduced apoptosis within kidney tissues following SA treatment (Fig. 5D). Systemic inflammatory responses were also suppressed, as evidenced by significant reductions in circulating TNF-α, IL-1β, and IL-6 concentrations (Fig. 5E). At the tissue level, qRT-PCR analysis confirmed that SA downregulated transcription of key proinflammatory cytokines and chemokines, including *Tnf-*α, *Il-1*β, *Il-6*, *Ccl2*, *Ccl3*, *and Cxcl2*, within the kidney (Fig. 5F). Collectively, these findings establish SA as a key fungal-derived metabolite capable of recapitulating the anti-inflammatory, renoprotective, and survival benefits conferred by *M. caribbica* during sepsis.

### SA suppresses macrophage activation via MAPK and NF-κB inhibition

**Fig 6:**
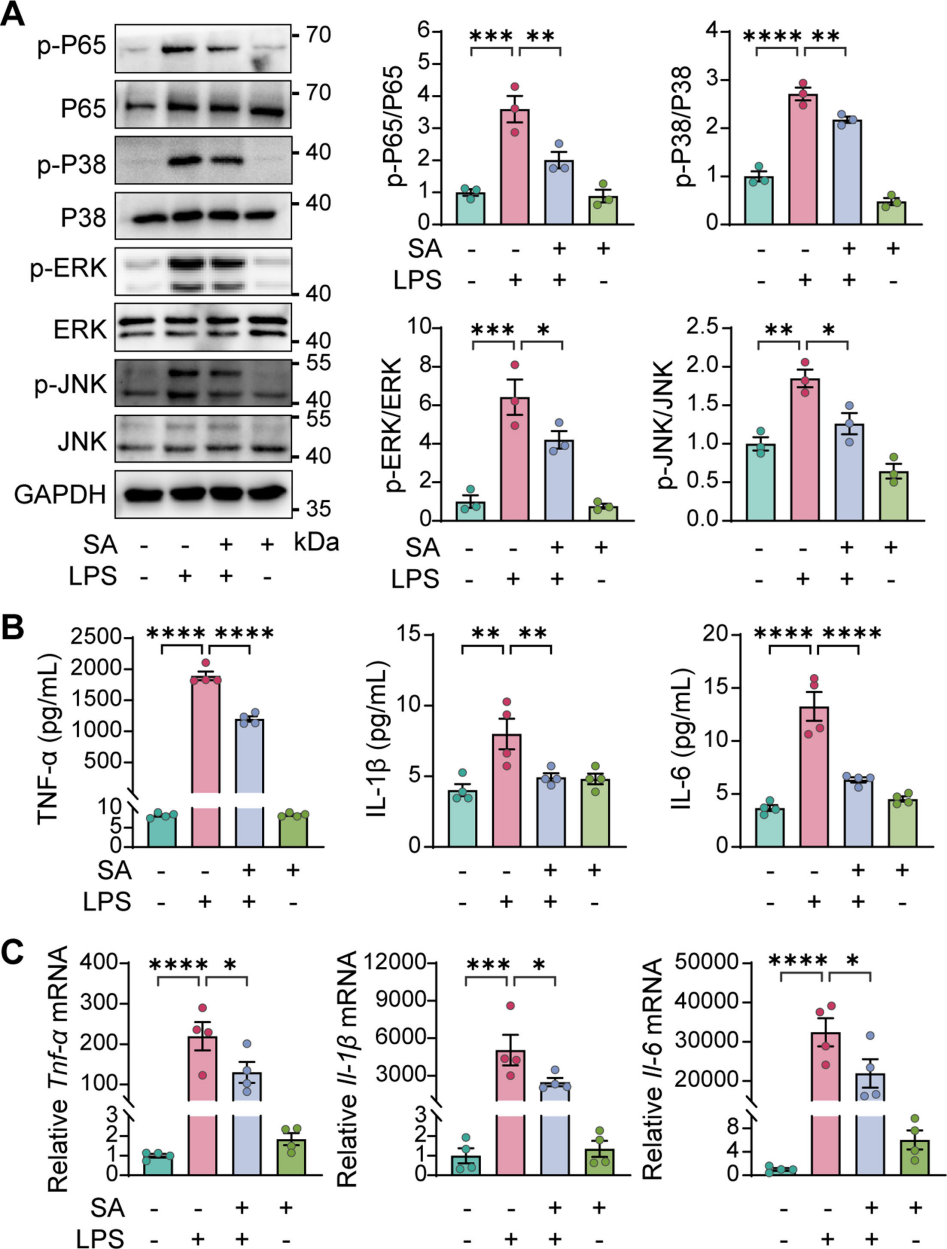
SA inhibits MAPK and NF-κB signaling in macrophages. (**A**) Western blot analysis of phosphorylated and total p65, p38, ERK1/2, and JNK in BMDMs pretreated with SA (10 μM, 2 h), followed by LPS stimulation (100 ng/mL, 30 min). *n* = 3. (**B**) ELISA quantification of TNF-α, IL-1β, and IL-6 levels in the culture supernatant of LPS-stimulated BMDMs with or without SA pretreatment. *n* = 4. (**C**) qRT-PCR analysis of *Tnf-*α, *Il-1*β, and *Il-6* mRNA expression in BMDMs treated as described above. *n* = 4. Data were represented as mean ± SEM. Statistical comparison was performed by one-way ANOVA with Holm-Sidak *post hoc* tests. *P < 0.05, **P < 0.01, ***P < 0.001, and ****P < 0.0001.

To investigate the molecular basis of SA-mediated anti-inflammatory effects, BMDMs were pretreated with SA prior to LPS stimulation. Western blot analysis revealed that SA markedly inhibited LPS-induced phosphorylation of core signaling molecules in the MAPK and NF-κB pathways, including p38, ERK1/2, JNK, and p65 (Fig. 6A). Consistently, SA pretreatment significantly reduced the secretion of TNF-α, IL-1β, and IL-6 (Fig. 6B), and suppressed the transcription of these cytokines, as confirmed by qRT-PCR (Fig. 6C). These results demonstrate that SA directly inhibits macrophage activation by inhibiting MAPK and NF-κB pathway phosphorylation, thereby dampening proinflammatory cytokine production and contributing to its systemic anti-inflammatory effects.

### SA inhibits macrophage pyroptosis by suppressing NLRP3 inflammasome activation

**Fig 7:**
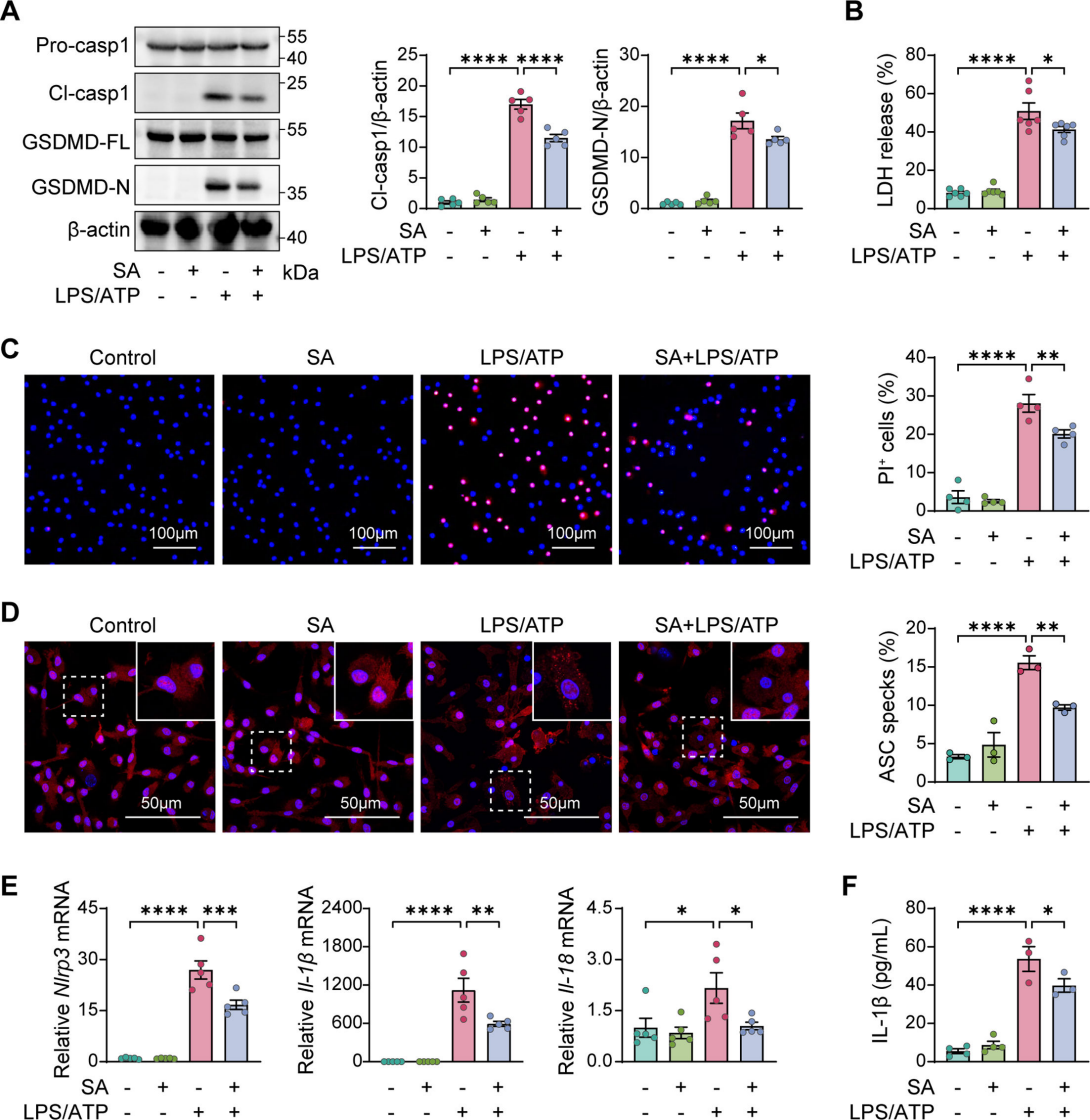
SA inhibits pyroptosis by suppressing inflammasome activation in macrophages. (**A**) Western blot analysis of cleaved caspase-1 (p20) and GSDMD-N in BMDMs pretreated with SA (10 μM, 2 h), followed by LPS (500 ng/mL, 2 h) and ATP (5 mM, 30 min). *n* = 5. (**B**) LDH release from BMDMs pretreated with SA (10 μM, 2 h), followed by LPS (500 ng/mL, 2 h) and ATP (5 mM, 1 h). *n* = 6. (**C**) Representative fluorescence images and quantification of propidium iodide (PI)-positive cells. *n* = 4. (**D**) Immunofluorescence staining of ASC specks and quantification of ASC-positive cells. *n* = 3. (**E**) qRT-PCR analysis of *Nlrp3*, *Il-1*β, and *Il-18* mRNA expression in BMDMs after LPS and ATP stimulation with or without SA pretreatment. *n* = 5. (**F**) ELISA quantification of IL-1β levels in the culture supernatant of BMDMs treated as above. *n* = 3 or 4. Data are presented as mean ± SEM. Statistical analysis was performed using one-way ANOVA with Sidak *post hoc* tests. *P < 0.05, **P < 0.01, ***P < 0.001, ****P < 0.0001. Scale bars: 50 or 100 μm.

Given the established role of macrophage pyroptosis in driving sepsis-associated inflammation and organ injury, we next assessed whether SA modulates inflammasome activation and pyroptotic cell death. In LPS-primed and ATP-stimulated BMDMs, SA markedly reduced the levels of caspase-1 p20 and the N-terminal fragment of GSDMD, indicating suppressed macrophage pyroptosis (Fig. 7A). Consistently, SA pretreatment significantly decreased LDH release (Fig. 7B) and reduced membrane permeability, as shown by diminished PI uptake (Fig. 7C). Immunofluorescence imaging revealed attenuated formation of ASC specks, a hallmark of NLRP3 inflammasome assembly (Fig. 7D). Moreover, SA downregulated *Nlrp3*, *Il1*β, and *Il18* mRNA expression (Fig. 7E) and reduced IL-1β secretion into the supernatant (Fig. 7F). Together, these results demonstrate that SA inhibits canonical pyroptosis by suppressing NLRP3 inflammasome activation, thereby limiting inflammatory cell death in macrophages.

## DISCUSSION

Sepsis is a life-threatening syndrome marked by immune dysregulation and multi-organ failure, with AKI as a common and severe complication strongly associated with poor prognosis (3, 24). The gut microbiota has emerged as a critical modulator of sepsis outcomes. In SAKI patients, *Clostridium difficile* colonization correlates with increased infection-related mortality (25). We recently demonstrated that pregnancy-induced gut microbiome shifts heighten susceptibility to sepsis in both mice and humans (26). Although the bacterial component of the microbiota has been well studied, the functional role of intestinal fungi remains insufficiently understood. Recent studies indicate that certain commensal fungi can functionally compensate for intestinal bacteria by maintaining mucosal integrity and modulating systemic immune responses (27). Fungal colonization or mannan supplementation reverses fluconazole-induced gut damage by inhibiting macrophage-derived serum amyloid A1 and reducing epithelial pyroptosis (28). However, the effects of fungi are context and species dependent. For example, in uremic mice, *Candida* overgrowth exacerbates intestinal and hepatic injury, whereas co-administration of *Lactobacillus rhamnosus* L34 mitigates disease severity (29). Together, these findings highlight the immunomodulatory potential of the gut mycobiome in shaping host responses during critical illness.

It is well known that fungi thrive in humid environments. Previous studies have shown that high-temperature and high-humidity conditions can reshape gut microbiota communities. For example, exposure to a hot and humid environment (31–33°C, 91–95% humidity) for 45 days induces gut dysbiosis and alters secondary bile acid metabolism, leading to anxiety-like behavior (30). Another study demonstrated that a hot and humid environment (32 ± 1°C, 85–90% humidity) for 14 days results in changes to the gut microbiota, such as reduced Bacteroidetes and increased Firmicutes (31). Furthermore, mice exposed to a hot and humid environment (32°C, 95% humidity) for 21 days showed significant alterations in their gut mycobiome, with a notable expansion of *Solicoccozyma aeria*, which was associated with appetite suppression and activation of IL-17 receptor signaling in the hypothalamic–gut–brain axis (32). Although prolonged exposure to hot and humid conditions has been associated with gut dysbiosis, our findings reveal that short-term exposure to high humidity at room temperature selectively reshapes the gut mycobiota, conferring protection against sepsis.

In this study, we investigated the impact of a 7-day preconditioning of mice under high-humidity conditions at room temperature. Our initial observations showed that this exposure extended survival after CLP-induced sepsis, reduced kidney injury, and alleviated systemic inflammation. Notably, these benefits persisted even after depletion of intestinal bacteria with ABX, but were abolished when intestinal fungi were eradicated using AMB. Given the broad-spectrum antifungal activity of AMB, the loss of protection strongly indicates that commensal fungi, rather than opportunistic pathogens, are responsible for mediating this effect. Furthermore, FMT from HH-exposed mice failed to reproduce the protective effect, suggesting that bacterial components alone may be insufficient to account for the observed protection. This aligns with recent findings indicating that fungal donor engraftment after FMT shows only weak associations with therapeutic outcomes, highlighting that fungal transfer during FMT is less efficient and more complex than bacterial transfer (33). It should therefore be noted that conventional FMT predominantly transfers bacterial communities, whereas fungal engraftment efficiency is limited. Thus, the absence of protection transfer via FMT does not fully exclude bacterial contributions but underscores the complexity of kingdom-specific effects. These data collectively identify gut fungi as key non-bacterial determinants of sepsis outcomes under altered humidity conditions.

To assess humidity-induced shifts in the gut mycobiome, we conducted ITS-based sequencing of fecal samples from mice housed under normal or high-humidity conditions at room temperature for 7 days. High humidity exposure significantly increased fungal richness and induced distinct community profiles, as evidenced by alpha diversity and PCoA analyses. LEfSe identified *Malassezia*, *Vishniacozyma*, *Sporobolomyces*, and *Meyerozyma* as key genera enriched under HH, with *Meyerozyma* emerging as a candidate of fungi due to its documented anti-inflammatory and probiotic properties in colitis models (17, 23). Subsequently, we confirmed the expansion of *M. caribbica* in HH-exposed mice with qRT-PCR. Oral administration of *M. caribbica* achieved stable colonization and recapitulated the protective effects of high humidity in conventional mice, improving survival, dampening systemic cytokine responses, and alleviating kidney injury in sepsis. These findings highlight *M. caribbica* as a humidity-responsive commensal with systemic immunomodulatory effects.

Given that microbial metabolites frequently mediate host–microbe interactions during sepsis, we conducted untargeted metabolomic analysis and identified SA as a metabolite associated with *M. caribbica* colonization. LC–MS/MS analysis confirmed SA enrichment in both fungal culture supernatants and cecal contents from *M. caribbica*-colonized or HH-exposed mice. SA is a naturally occurring phenolic compound broadly distributed in plant-derived foods and traditional Chinese medicines, and is known to exert diverse biological effects, including cardioprotective, anti-inflammatory, anti-cancer, and neuroprotective activities (34). SA can be microbially produced by bacteria or fungi through fermentation or biotransformation (18, 35, 36), or further metabolized and degraded by microbial pathways (37). In our study, short-term high humidity exposure significantly increased *M. caribbica* abundance and enhanced SA production. SA is a phenolic compound that may arise from microbial biotransformation of dietary lignin-derived phenolics or aromatic amino acid metabolites (38). Yeasts are known to harbor enzymes involved in phenolic acid metabolism (39), suggesting that *M. caribbica* may generate SA through specific oxidative or decarboxylation pathways. However, the precise enzymatic mechanisms require further investigation.

SA exerts immunomodulatory effects on macrophages, the key drivers of early hyperinflammatory responses during sepsis. Its ability to dampen LPS-induced transcription and secretion of proinflammatory cytokines suggests a broad anti-inflammatory potential. Mechanistically, the inhibition of p38, ERK1/2, JNK, and p65 phosphorylation indicates that SA interferes with both MAPK and NF-κB signaling cascades—pathways central to macrophage activation. Moreover, given the critical contribution of pyroptosis to sepsis progression (40), our findings indicate that SA also mitigates inflammasome-driven macrophage pyroptosis. Specifically, SA attenuates canonical pyroptosis through suppression of caspase-1 and GSDMD cleavage, disruption of inflammasome assembly, and reduction of IL-1β and IL-18 expression. These effects collectively reflect decreased membrane rupture and reduced inflammatory amplification.

However, our study has several limitations. First, the enzymatic pathway responsible for SA biosynthesis in *M. caribbica* remains to be elucidated. Second, the contribution of other fungal metabolites or interkingdom microbial interactions cannot be fully excluded. Third, our *in vitro* data support a macrophage-mediated mechanism for SA, but further *in vivo* studies assessing the impact on macrophage function and related outcomes will be necessary to confirm these findings. In addition, although immune-mediated renal protection is supported, the direct effects of SA on renal tubular epithelial cells were not systematically examined and warrant further investigation. Finally, validation in human samples or clinically relevant models is needed to establish translational significance. Future studies integrating multi-omics will help clarify the causal relationship between *M. caribbica*–derived metabolites and host immune regulation during sepsis.

In conclusion, our study identifies *M. caribbica* as a beneficial intestinal fungus enriched after short-term exposure to high humidity conditions at room temperature. SA is revealed as a protective fungal metabolite that mitigates inflammation, pyroptosis, and kidney injury during sepsis. These findings underscore the importance of environmental modulation of the gut mycobiome in host immunity and highlight the therapeutic potential of fungal-derived metabolites in critical illness.

## Data Availability

Data will be made available upon request. The raw data of the ITS sequencing have been deposited in the CNGB database under accession number CNP0008351, and the metabolomics data have been deposited under accession number CNP0008340.

## SUPPLEMENTAL MATERIAL

Table S1 msystems.01716-25-s0001.xlsx Differential metabolites identified between *M. caribbica* culture supernatant and YM basal medium by LC-MS-based untargeted metabolomics analysis.

ASM does not own the copyrights to Supplemental Material that may be linked to, or accessed through, an article. The authors have granted ASM a non-exclusive, world-wide license to publish the Supplemental Material files. Please contact the corresponding author directly for reuse.
